# Genome Sequence of Equid Alphaherpesvirus 1 (EHV-1) from a Nasal Swab of a Swiss Horse Associated with a Major EHV-1 Outbreak following a Show Jumping Event in Valencia, Spain

**DOI:** 10.1128/MRA.00732-21

**Published:** 2021-08-26

**Authors:** Jakub Kubacki, Julia Lechmann, Cornel Fraefel, Claudia Bachofen

**Affiliations:** a Institute of Virology, University of Zurich, Zurich, Switzerland; Portland State University

## Abstract

We present the genome sequence of equid alphaherpesvirus 1 (EHV-1) sequenced directly from the nasal swab of a Swiss horse that attended an international equestrian event in Valencia, Spain, the origin of an outbreak of neurological disorders in horses in several European countries in February 2021.

## ANNOUNCEMENT

In February 2021 during the CES Valencia Spring Tour, an outbreak of equid alphaherpesvirus 1 (EHV-1) was reported. Since then, 18 horses have died, and cases of EHV-1 linked to the outbreak have been confirmed in 10 countries (https://inside.fei.org/fei/ehv-1).

An 11-year-old gelding presented with fever but without neurological signs after returning from Valencia, Spain, and a nasal swab was collected (25 February) by a private veterinarian. The DNA was isolated using the QIAamp DNA minikit (Qiagen) and eluted in 100 μl of elution buffer. An EHV-1/4 multiplex real-time PCR (1, 2) showed the sample to be positive for EHV-1 (threshold cycle [*C_T_*] value, 23) but not EHV-4. A sequencing library of the same DNA was prepared using the NEBNext Ultra II DNA library prep kit and the NEBNext multiplex oligos for Illumina (96 unique dual index primer pairs; New England BioLabs) and sequenced on the Illumina NovaSeq system in a paired-end 2 × 150-nucleotide (nt) run at the Functional Genomics Centre Zurich (FGCZ; Zurich, Switzerland). In total, 144 million raw reads were generated and analyzed using default settings of two in-house pipelines. Sequencing adaptors and low-quality ends were trimmed from quality-controlled reads (FastQC v0.11.17) (average quality lower than 20 within a 4-nt window) using Trimmomatic v0.36 (3). Then, reads were *de novo* assembled using MEGAHIT v1.1.3 and metaSPAdes v3.12 (4, 5). Subsequently, *de novo* assembled contigs and quality-controlled reads were realigned to all available full-length EHV-1 GenBank references, including the five EHV-1 strains sequenced from Belgian and French horses that attended the same event in Valencia, Spain (6), using a metagenomic pipeline of the SeqMan NGen v17 (Lasergene; DNAStar, USA). Sanger sequencing of three 500- to 800-nt-long fragments was performed to bridge selected gaps and confirm the Illumina-based sequence (Table 1).

**TABLE 1:** Overview of the primer pairs used to bridge selected sequence gaps by Sanger sequencing

Primer	Sequence 5′–3′	Start^*a*^	End^*a*^	Product length (nt)
EHV1_A2_f	TTTCGGCCTCGCGACGGCCTCGAAG	41	65	739
EHV1_A2_r	TGTCGCGGAGCGGGTTGAACG	760	780	
EHV1_C_f	CCTCGTCGGACGACATTGTG	41255	41274	581
EHV1_C_r	GGTCAAACGACGCCAAGAGG	41817	41836	
EHV1_N_f	ACTCGGCTGATGCGCAATG	131791	131815	811
EHV1_N_r	TATCCTGGCGTCCTCGAAC	132585	132602	

*^a^* Position relative to MZ357402.

A near-complete genome sequence of EHV-1 with a length of 151,306 nt (GC content, 56.6%; average depth, 3.92×) covering 96% (SeqMan) of the NCBI reference sequence (GenBank accession number NC_001491.2) was generated from 3,961 reads. With 99.8% nucleotide identity (Blastn v2.12), the sequence is most closely related to strains from Belgium and France (MW855958.1, MW855959.1, MW855960.1, MW855961.1, and MW855962.1) and, as expected, also belongs to clade 10 (6, 7). Like the other strains from the outbreak, it lacks the point mutation at amino acid position 752 of the polymerase, frequently associated with neuropathogenicity (8). Remarkably, while other strains from this outbreak were sequenced after virus isolation and using the Oxford Nanopore technology, sequencing directly from extracted DNA of a nasal swab sample using the Illumina system was successful. While this may have contributed to the differences observed between this sequence and the Belgian and French strains, it shows that even without prior virus isolation, it is possible to generate a near-full-length EHV-1 genome sequence, allowing in-depth phylogenetic analysis and molecular tracing also in cases where virus isolation is not possible or not successful.

### Data availability.

The sequence has been deposited in GenBank under the accession number MZ357402. The raw data have been deposited in the NCBI Sequence Read Archive under the accession number SRX11352325.
